# Rescue of Eosinophilic Cardiac Tamponade by Anakinra in a Pregnant Woman With Familial Mediterranean Fever

**DOI:** 10.1155/crii/1244531

**Published:** 2026-08-25

**Authors:** Maxime Quaggetto, Matthieu Groh, Lea Savey, Philippe Rouvier, Quentin Moyon, Sophie Georgin-Lavialle, Marc Pineton de Chambrun

**Affiliations:** ^1^ Sorbonne Université, Assistance Publique-Hôpitaux de Paris (AP-HP), Service de Médecine-Intensive Réanimation, Institut de Cardiologie, Hôpital La Pitié-Salpêtrière, Paris F-75013, France, aphp.fr; ^2^ Sorbonne Université, Assistance Publique-Hôpitaux de Paris (AP-HP), Service de Médecine Interne 2, Institut E3M, Hôpital La Pitié-Salpêtrière, Paris F-75013, France, aphp.fr; ^3^ Sorbonne Université, INSERM, CIMI-Paris, U1135, Hôpital La Pitié-Salpêtrière, Paris F-75013, France, aphp.fr; ^4^ Centre de Référence des Syndromes Hyperéosinophiliques (CEREO), Service de Médecine Interne, Hôpital Foch, Suresnes F-92150, France, hopital-foch.org; ^5^ Sorbonne Université, Assistance Publique-Hôpitaux de Paris (AP-HP), Service de Médecinte Interne, Hôpital Tenon, Paris F-75020, France, aphp.fr; ^6^ Centre de Référence des Maladies Auto-inflammatoires et des Amyloses Inflammatoires (CEREMAIA), Hôpital Tenon, Paris F-75020, France, aphp.fr; ^7^ Sorbonne Université, Assistance Publique-Hôpitaux de Paris (AP-HP), Service d’Anatomopathologie, Hôpital La Pitié-Salpêtrière, Paris F-75013, France, aphp.fr

**Keywords:** familial Mediterranean fever, hypereosinophilic syndrome, myocarditis, pericarditis

## Abstract

Familial Mediterranean fever (FMF) is an interleukin‐1 (IL‐1)‐driven autoinflammatory disease in which pericarditis, occasionally complicated by cardiac tamponade, is a recognized manifestation, whereas hypereosinophilia is not. We report a woman in her thirties with FMF (homozygous *MEFV* M694V mutation) receiving colchicine 2.5 mg/day, admitted in the third trimester of pregnancy for dyspnea, fever, and cough. She had blood hypereosinophilia (2.9 G/L), bilateral lung consolidation with bronchoalveolar lavage eosinophilia (46%) and myopericarditis. Cardiac tamponade required emergency pericardiocentesis and cesarean section, with favorable maternal and neonatal outcomes; pericardial fluid contained more than 90% eosinophils. Infectious, autoimmune, drug‐induced, and clonal causes were excluded. High‐dose corticosteroids only transiently reduced the eosinophil count, which rebounded, while C‐reactive protein remained elevated. Both parameters normalized after anakinra was started, allowing corticosteroid withdrawal, with sustained remission at 36 months. Whether hypereosinophilia was a manifestation of FMF or a coincidental association remains undetermined. IL‐1 blockade nonetheless controlled both the serositis and the eosinophilia, and may limit corticosteroid exposure.

**Summary**

• Hypereosinophilic syndrome (HES) is not a common feature of familial Mediterranean fever (FMF). This rare and potentially life‐threatening condition, when associated with FMF, can lead to severe cardiac complications but may respond effectively to interleukin‐1 (IL‐1) inhibition with anakinra.

## 1. Introduction

Familial Mediterranean fever (FMF) is a monogenic autoinflammatory disease driven by dysregulated innate immunity and interleukin‐1 (IL‐1) signaling. Serosal involvement is common, and pericarditis—occasionally complicated by cardiac tamponade—is a recognized manifestation of FMF [1]. In contrast, hypereosinophilia is not considered a typical feature of FMF. However, eosinophilic complications have been reported in autoinflammatory conditions, including adult‐onset Still’s disease, suggesting potential overlap between IL‐1‐mediated disease and eosinophil‐mediated tissue injury [2]. We report a case of acute eosinophilic myopericarditis and pneumonitis in a pregnant woman with genetically confirmed FMF, highlighting a rare and poorly characterized association between FMF and hypereosinophilic disease.

## 2. Case Report

We present a rare case of hypereosinophilic syndrome (HES) associated with FMF in a pregnant woman in her thirties, diagnosed at 2 years of age (homozygous *MEFV* M694V mutation) and receiving daily colchicine (2.5 mg). She had no history of asthma, allergy, atopic dermatitis, or chronic rhinosinusitis with nasal polyposis. Her family originated from Tunisia, and this was her first pregnancy. Her attacks were typically triggered by fatigue and psychological stress, with no identified infectious or menstrual trigger. Over the 6 months preceding the admission and on 2.5 mg of colchicine daily, she had experienced three attacks, one of which involved chest pain. In her third trimester, she presented to the emergency department with dyspnea, preceded by 1 week of myalgia, fever, diarrhea, and cough, for which she received amoxicillin for 2 days. Physical examination revealed tachypnea, orthopnea, and bilateral lung crackles without typical FMF features. Oxygen saturation was 90% on room air, requiring high‐flow nasal oxygen after ICU admission. Laboratory tests showed leukocytosis (14 G/L) with hypereosinophilia (2.9 G/L), troponin elevation (113 ng/L, ULN 50 ng/L), and normal CRP. ECG showed sinus tachycardia, and echocardiography revealed a 6‐mm pericardial effusion. Chest CT revealed bilateral lung consolidation without pulmonary embolism. Bronchoalveolar lavage showed marked eosinophilia (46%) with negative microbiological findings. Cefotaxime and levofloxacin were started for suspected bacterial pneumonia. Pericardial effusion progressed to 15‐mm, LVEF decreased to 45%, and troponin rose to 370 ng/L. Cardiac MRI showed lateral and apical myocardial edema and a 12 mm pericardial effusion. After 6 days in the ICU, cardiac tamponade occurred with a decrease in LVEF (40%) on echocardiography, leading to emergency pericardiocentesis and cesarean section due to fetal distress. Delivery occurred at 31 weeks (birth weight 1630 g, Apgar score 10) with favorable maternal and neonatal outcomes.

**Figure 1:**
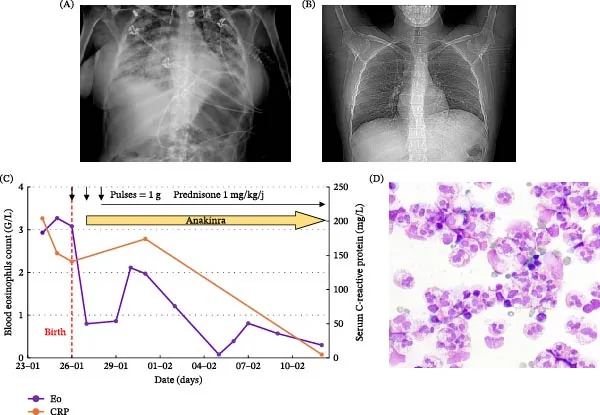
Main clinical and laboratory findings and therapeutic interventions in a woman with life‐threatening eosinophilic cardiac tamponade associated with FMF. (A, B) Chest radiograph before and after pericardiocentesis. (C) Eosinophil count and CRP during the ICU stay, showing the transient effect of corticosteroid pulses on the eosinophil count with subsequent rebound, persistently elevated CRP, and sustained normalization of both parameters after anakinra initiation. (D) Cytological analysis of the pericardial fluid showing a clear predominance of eosinophils.

Pericardial fluid analysis showed >90% eosinophils. Colchicine was discontinued due to persistent inflammatory activity on treatment, and methylprednisolone pulses (1 g for 3 days) followed by oral prednisone (1 mg/kg) were started. Anakinra (100 mg/day) was initiated because of persistent inflammation (CRP 174 mg/L) suggestive of an FMF flare, concurrently with a rebound in the eosinophil count after corticosteroid pulses. Both parameters normalized thereafter, and she was discharged after 3 weeks (Figure 1). Corticosteroids were tapered and withdrawn over 6 months. Anakinra was switched to canakinumab at 6 months because of poor tolerance of daily subcutaneous injections. Colchicine was resumed and maintained at 1 mg/day. At the last follow‐up, 36 months after the index episode, the patient remained in remission with fewer than three attacks per year and a normal eosinophil count. The final diagnosis was acute eosinophilic myopericarditis and pneumonitis of unknown origin after exclusion of infectious, autoimmune, drug‐induced and clonal eosinophilia.

We investigated the association between FMF and hypereosinophilia through a literature search of MEDLINE, Embase, and the Cochrane Library. The search was limited to English‐language articles published between 2000 and 2024 and used the keywords “familial Mediterranean fever,” “MEFV,” “FMF,” “eosinophil,” “eosinophilic,” and “hypereosinophilia.” Of the 192 articles retrieved, 38 were duplicates and 140 were irrelevant, leaving 14 articles covering 20 patients (Table S1).

## 3. Discussion

HES is a rare association with FMF, typically affecting the gastrointestinal tract. A previous case of pericarditis with fever and hypereosinophilia, involving nonclassical MEFV variants (E84K and G304R), led to an FMF diagnosis without confirmation of eosinophilic pericardial involvement; hypereosinophilia nevertheless responded to colchicine [1]. A final “incomplete type” of FMF diagnosis was made according to the Tel‐Hashomer criteria [3]. Few cases of eosinophilic pneumonia or asthma have been reported, but none with an acute onset [4, 5]. Eosinophilic diseases are also described in other autoinflammatory disorders, such as Cryopyrin‐associated periodic syndrome [4] and Still’s disease [2, 6]. Whether hypereosinophilia represents a manifestation of FMF or a coincidental association remains unresolved, as eosinophilia is not part of the diagnostic criteria for FMF. In our patient, colchicine at 2.5 mg/day represented the ceiling of monotherapy, and three attacks over the preceding 6 months indicated that disease control nonetheless remained incomplete. High‐dose corticosteroids induced only a transient decrease in the eosinophil count, which rose again over the following days, while CRP remained elevated (174 mg/L; Figure 1C). Sustained normalization of both the eosinophil count and CRP was obtained only after the introduction of anakinra, a response comparable to that reported in colchicine‐resistant FMF and in idiopathic (autoinflammatory) recurrent pericarditis. The concomitant resolution of the systemic inflammatory response and of the eosinophilia during IL‐1 blockade is compatible with a shared IL‐1‐dependent mechanism, although the concurrent corticosteroid therapy and the single‐patient report preclude any firm causal inference.

## 4. Conclusion

Whether hypereosinophilia was a manifestation of FMF or a coincidental association could not be established. Despite this uncertainty, IL‐1 blockade controlled both the serosal inflammation and eosinophilia, allowed corticosteroid withdrawal, and maintained remission at 36 months. The overlap between IL‐1‐driven autoinflammation and eosinophil‐mediated tissue injury supports considering anti‐IL‐1 therapy in similar presentations, with the potential benefit of limiting corticosteroid exposure.

## Funding

No funding was received for this study.

## Disclosure

All authors had access to the data and significantly contributed to study design, data collection, manuscript drafting, critical evaluation, and/or final approval.

## Consent

The patient provided written informed consent for the publication of her clinical details and associated images.

## Conflicts of Interest

The authors declare no conflicts of interest.

## Supporting Information

Additional supporting information can be found online in the Supporting Information section.

## Supporting information


**Supporting Information** Table S1: Cases of MEFV mutations‐related diseases associated to hypereosinophilic disorders.

## Data Availability

The data that support the findings of this study are available upon request from the corresponding author. The data are not publicly available due to privacy or ethical restrictions.
