# Supplementary material for: Rescue of Eosinophilic Cardiac Tamponade by Anakinra in a Pregnant Woman With Familial Mediterranean Fever
Source: Case Reports Immunol. 2026 Aug 25;2026:1244531. doi: 10.1155/crii/1244531 (PMC13505191; doi:10.1155/crii/1244531)
Supplement: Supplementary file 1 [file CRII-2026-1244531-s001.docx]

|  | **Supplemental Table 1. Cases Of MEFV** **Mutations-Related Diseases Associated To Hypereosinophilic Disorders** | | | | | | | |
| --- | --- | --- | --- | --- | --- | --- | --- | --- |
|  | **Journal** | **Year** | **First author** | **MEFV-related disease** | **Age** | **Sex** | **Organ involvement** | **Highest Blood Eosinophils** |
|  | *South Med J* | 2010 | Kocak G | FMF | 39 | M | Colitis | 3.3 G/L |
|  | *Intern Med* | 2014 | Yoshioka K | “FMF-like”^1^ | 56 | M | Pericarditis, pleuritis, skin | 2.5 G/L |
|  | *Indian J Hematol Blood Transfus* | 2014 | Keklik M | FMF | 27 | F | None | 5 G/L |
|  | *Mod Rheumatol* | 2015 | Kushima H | FMF | 49 | F | Necrotizing lung nodules, pleuritis | Over ULN |
|  | *Case Rep Gastrointest Med* | 2017 | Rohani P | FMF | 3 | F | Esophagitis | Under ULN |
|  | *Intern Med J* | 2017 | Jang HS | FMF | 42 | F | Esophagitis, monoclonal IgE | Under ULN |
|  | *Turk J Pediatr* | 2017 | Çakr M | FMF | n/a | n/a | Gastroenteritis | n/a |
|  | *Acta Med Iran* | 2019 | Mehrabani S | FMF | 13 | F | Gastroenteritis | n/a |
|  | *Ann Rheum Dis* | 2019 | Hong Y | PAAND | 12 | M | Oral ulceration, lymph node, skin, gastroenteritis | 82 G/L |
|  | *Ann Rheum Dis* | 2019 | Hong Y | PAAND | 9 | M | Oral ulceration, lymph node, lung, skin, gastroenteritis | 82 G/L |
|  | *Ann Rheum Dis* | 2019 | Hong Y | PAAND | 2 | M | Oral ulceration, osteitis | Over ULN |
|  | *Pediatr Int* | 2022 | Salman H | FMF | 4 | F | Gastroenteritis | Under ULN |
|  | *Int Arch Allergy Immunol* | 2022 | Üsküdar Cansu D | 5 FMF | n/a | n/a | n/a | > 1.5 G/L |
| *Allergy Asthma Clin Immunol* | | 2023 | Alotaibi BM | FMF | 56 | F | Asthma, lymph node, skin | 0.7 G/L |
|  | *Isr Med Assoc J* | 2023 | Wand O | FMF | 63 | F | Pneumonia | Under ULN |
|  | **Abbreviation :** DRESS, drug rash with eosinophilia and systemic symptoms; FMF, familial mediterranean fever; HES, hypereosinophilic syndrome; MEFV, Mediterranean fever; PAAND, pyrin-associated autoinflammation with neutrophilic dermatosis; ULN, upper limit of normal value.  ^1^ Two variants of the MEFV gene, E84K and G304R are not clearly linked with the FMF phenotype. The diagnosis was made through Tel-Hashomer criteria, leading to an “incomplete type” of FMF. | | | | | | | |
